# *Rehmannia glutinosa* nanovesicles protect cardiomyoblasts from oxidative injury

**DOI:** 10.20517/evcna.2025.185

**Published:** 2026-04-22

**Authors:** Huilan Fan, Shuang Zhao, Yunfeng Di, Yangyi Yu, Yafei Sun, Yong Wang, Chun Li, Jingyu Wang

**Affiliations:** ^1^College of Traditional Chinese Medicine, Beijing University of Chinese Medicine, Beijing 102488, China.; ^2^Dongzhimen Hospital, Beijing University of Chinese Medicine, Beijing 100007, China.; ^3^Key Laboratory of Traditional Chinese Medicine Syndrome and Formula, Ministry of Education, Beijing 100029, China.; ^4^Modern Research Center for Traditional Chinese Medicine, Beijing University of Chinese Medicine, Beijing 100029, China.

**Keywords:** Plant-derived nanovesicles, *Rehmannia glutinosa*, myocardial injury, oxidative stress, phytomedicine

## Abstract

**Aim:** Oxidative stress is a key driver of cardiovascular disease, underscoring the need for safe and effective antioxidant therapies. This study aims to evaluate the cardioprotective potential of plant-derived nanovesicles (PDNVs) derived from *Panax ginseng* (Gin) and *Rehmannia glutinosa* (Glu) against hydrogen peroxide (H_2_O_2_)-induced oxidative injury in cardiac cells.

**Methods:** PDNVs were isolated from medicinal plants via differential ultracentrifugation and characterized for morphology, diameter, stability, and cellular uptake. The antioxidant and cytoprotective effects were assessed in H_2_O_2_-injured cardiomyoblasts through cell viability, 2,2-diphenyl-1-picrylhydrazyl radical (DPPH·) radical scavenging, intracellular reactive oxygen species (ROS) and mitochondrial superoxide detection, and antioxidant enzyme (superoxide dismutase, SOD; glutathione peroxidase, GPx) activity assays. The composition of the PDNVs was determined through Coomassie Brilliant Blue staining for proteins, agarose gel electrophoresis for nucleic acids, and liquid chromatography-mass spectrometry system (LC-MS) for bioactive monomers.

**Results:** Gin-PDNVs and Glu-PDNVs significantly enhanced cardiomyoblast viability under oxidative stress. Glu-PDNVs demonstrated superior efficacy at lower concentrations, with stronger ROS scavenging capacity. Compositional analysis revealed that Glu-PDNVs carry proteins, nucleic acids, and antioxidant herbal compounds such as catalpol, rehmannioside D, and acteoside. Glu-PDNVs also dose-dependently scavenged DPPH· radicals, reduced mitochondrial superoxide accumulation, and significantly restored the H_2_O_2_-induced suppression of SOD and GPx activities.

**Conclusion:** This study provides the first evidence that Glu-PDNVs exert potent cardioprotection by regulating ROS and superoxide homeostasis, positioning them as a promising natural nanotherapeutic platform with translational potential.

## INTRODUCTION

The extensive occurrence of redox reactions makes oxidative stress a critical factor affecting cardiac functions. Oxidative stress is defined as an imbalance where the body’s antioxidant defense system is insufficient to eliminate pro-oxidative molecules (such as reactive oxygen species, ROS), leading to oxidative modifications of key biomolecules such as proteins, lipids, and nucleic acids^[1]^. Such molecular damage can disrupt mitochondrial function and induce apoptosis and ferroptosis^[2-4]^. Ultimately, it triggers adverse cardiac remodeling in various heart diseases including myocardial infarction, cardiac ischemia-reperfusion injury, and heart failure^[5,6]^. Consequently, developing highly effective, biocompatible antioxidant therapeutics has become a critical focus of contemporary cardiovascular research. Cell-derived nanovesicles (CDVs) are lipid bilayer-structured particles possessing excellent biocompatibility and low immunogenicity in the delivery of bioactive molecules such as proteins and lipids from donor cells to recipient cells, thereby regulating the physiological functions of recipient cells^[7,8]^. Current research has focused extensively on CDVs derived from animal cells. Among these, mesenchymal stromal cell-derived extracellular vesicles (MSC-EVs) exhibit significant antioxidant properties and can regulate cellular processes such as proliferation, apoptosis, inflammation, and angiogenesis. Consequently, they show promising therapeutic potential in the treatment of various cardiovascular diseases^[9-11]^. However, MSC-EVs suffer from inherent limitations such as low yield and high cost, which severely hinder their clinical translation process^[12-14]^.

Research has revealed that nanovesicles extracted from plants are similar to animal CDVs in their structure, content, and release mechanisms^[15]^. These are called plant-derived nanovesicles (PDNVs) and typically range from 30-200 nm^[16,17]^. PDNVs exhibit excellent biocompatibility, comparable to nanovesicles derived from animal cells. Moreover, studies have shown that PDNVs are efficiently internalized by recipient cells and do not induce significant cytotoxicity^[18]^. Importantly, PDNVs lack mammalian antigens and display lower immunogenicity^[19]^. In animal model experiments, PDNV administration did not cause detectable pathological damage to major organs or abnormalities in key blood biochemical parameters, indicating negligible systemic toxicity^[20]^. Moreover, their raw materials are abundant and do not require costly *in vitro* culture systems, enabling more efficient and economical large-scale production. PDNVs provide unique therapeutic advantages by preserving active herbal components and delivering superior pharmacological effects compared to the raw herbs^[21-23]^. Their lipid structure protects bioactive compounds from gastric and intestinal degradation, greatly enhancing oral bioavailability^[24,25]^. Oral administration is convenient and improves patient compliance, while other routes, such as intraperitoneal, intravenous, or intranasal delivery, and biomaterial-based cardiac patches, can further optimize biodistribution and targeting^[26-30]^. Given these advantages, PDNVs derived from medicinal herbs have shown promising antioxidant and cardioprotective activities. For instance, *Panax ginseng* (Gin)-PDNVs alleviate hydrogen peroxide (H_2_O_2_)-induced damage in Human Umbilical Vein Endothelial Cells (HUVECs) and treat cisplatin-induced cardiac injury by suppressing oxidative stress^[31,32]^. Similarly, *Salvia miltiorrhiza*-PDNVs have been demonstrated to promote angiogenesis, thereby improving myocardial ischemia-reperfusion injury^[33]^.

Among medicinal plants with therapeutic potential, *Rehmannia glutinosa* (Glu) stands out due to its well-established antioxidant and cardioprotective properties. As a plant with both medicinal and food uses, Glu contains bioactive constituents, e.g., catalpol, rehmannioside D, acteoside, and polysaccharides, which have demonstrated pharmacological effects^[34-37]^. Catalpol, the most abundant iridoid glycoside, serves as the official quality control marker in the Chinese Pharmacopoeia, while rehmannioside D and acteoside are critical for species authentication. However, the clinical translation of these bioactive compounds faces challenges including poor stability, low solubility, and limited oral bioavailability^[38,39]^. Meanwhile, the application of its polysaccharides is hindered by a lack of efficient preparation methods, unclear structure-activity relationships, and insufficient *in vivo* evidence^[40]^. In contrast, PDNVs not only efficiently concentrate bioactive constituents but also feature straightforward preparation, structural stability, and significantly enhanced bioavailability.

Based on a H_2_O_2_-induced rat H9c2 cardiomyoblast injury model, this study assessed the antioxidant capacity of Glu-PDNVs in comparison with Gin-PDNVs. Furthermore, we investigated the regulatory effects of Glu-PDNVs on intracellular ROS and superoxide levels, as well as on the activities of the antioxidant enzymes superoxide dismutase (SOD) and glutathione peroxidase (GPx). This study will provide provide new support for developing herbal PDNV-based antioxidant strategies for cardiac protection.

## METHODS

### Cell culture and H_2_O_2_ injury model

H9c2 cells, a cardiomyoblast cell line, were kindly provided by Yang Lu and Professor Shuwen Guo at Beijing University of Chinese Medicine^[41]^. These cells were maintained at 37 °C with 5% CO_2_ and saturated humidity. The complete medium used was prepared with 4.5 g·L^-1^ high-glucose Dulbecco’s Modified Eagle Medium (DMEM) medium (Gibco, Grand Island, NY, USA), supplemented with 10% fetal bovine serum (FBS; Procell, Wuhan, China) and 1% penicillin-streptomycin solution (P/S; Gibco, Grand Island, NY, USA). The medium was replaced every two days, and all experiments used cells in the logarithmic growth phase. Following a 24 h adhesion period post-seeding, H9c2 cells were subjected to oxidative stress by treatment with H_2_O_2_ (MREDA, Beijing, China) in serum-free, high-glucose (4.5 g·L^-1^) DMEM for 4 h. Subsequently, the cells were returned to complete medium and cultured for an additional 20 h.

### Isolation of PDNVs

Gin and Glu were purchased from Ruifeng Beiling Chinese Herbal Medicine Co., Ltd. in Anguo City. The plant materials were authenticated by Wei Li at Beijing University of Chinese Medicine according to pharmacopeial standards. After washing, the herbs were crushed to extract juice. The resulting herbal solution was filtered through gauze, and PDNVs were subsequently isolated from the filtrate using differential ultracentrifugation (Thermo, Waltham, MA, USA) at 4 °C. Centrifugation was performed sequentially at 300 × *g* for 20 min, 2,000 × *g* for 30 min and 12,000 × *g* for 60 min to remove insoluble debris. The supernatant was centrifuged again at 100,000 × *g* for 70 min. The resulting pellet was resuspended in phosphate-buffered saline (PBS; HyClone, Logan, UT, USA) and filtered through a 0.22 µm polyethersulfone membrane. The final PDNVs were analysed for protein concentration using the BCA Protein Assay Kit (Applygen, Beijing, China). The PDNV dosage concentration used in this study corresponds to the protein concentration.

### Transmission electron microscopy

A small amount of sample was pipetted onto the copper grid surface. After standing at room temperature for 10 min, residual liquid was blotted off with filter paper. Subsequently, the sample was counterstained with 3% uranyl acetate solution. The staining was performed at room temperature for 1-3 min. After allowing the sample to air dry, its morphological features were observed and analyzed using a transmission electron microscope (TEM; Hitachi, Tokyo, Japan).

### Nanoparticle tracking analysis and zeta potential

The PDNV samples were appropriately diluted with PBS buffer. The particle size distribution, concentration, and zeta potential of the PDNVs were measured using a ZetaView PMX-X30 nanoparticle analyzer (Particle Metrix, Meerbusch, Germany).

### Chlorophyll content assay

Chlorophyll content was measured to assess potential chloroplast contamination in PDNV preparations. Samples were mixed with an equal volume of absolute ethanol, incubated at 4 °C in the dark for 20-30 min, and then centrifuged at 12,000 rpm for 5 min. The supernatant was collected, and absorbance at 663 and 645 nm, corresponding to chlorophyll a and b, respectively, was measured using a microplate reader. Spinach homogenate and PBS served as positive and negative controls, respectively. Chlorophyll content was detected by absorbance at 663 and 645 nm.

### Detection of cargo in Glu-PDNVs

To comprehensively characterize the molecular composition of PDNVs, a multi-faceted analysis was performed. Nucleic acids were extracted using TRIzol reagent (Thermo, Waltham, MA, USA), quantified via Nano Drop spectrophotometry (Implen, Munich, Germany), and assessed by Gel Red-stained agarose gel electrophoresis (Vazyme, Nanjing, China; baygene, Shanghai, China). Concurrently, PDNV proteins were separated by Sodium Dodecyl Sulfate Polyacrylamide Gel Electrophoresis (SDS-PAGE) and visualized with Coomassie Brilliant Blue staining (Epizyme, Shanghai, China; sparkjade, Shandong, China). For the analysis of bioactive small molecules, PDNVs were first disrupted by methanol treatment and ultrasonication. The resulting extracts, collected after centrifugation (12,000 rpm, 8 min), were analyzed using a 1290 II - 6460 liquid chromatography-mass spectrometry system (LC-MS; Agilent Technologies, Santa Clara, CA, USA). The reference standards were catalpol, rehmannioside D, and acteoside [High-Performance Liquid Chromatography (HPLC) ≥ 98%; Yuanye, Shanghai, China].

### Cell viability assay using CCK-8

The c assay (Vazyme, Nanjing, China) was employed to assess cell viability according to the provided instructions. H9c2 cells were simultaneously treated with H_2_O_2_ and PDNVs in FBS-free DMEM for 4 h, followed by replacement with complete medium for an additional 20 h of culture. Cell Counting Kit-8 (CCK-8) reagent was then added to the cells, which were incubated at 37 °C in a 5% CO_2_ environment for 2 h. The absorbance at 450 nm was detected using a microplate reader.

### ImageXpress pico automated cell imaging and analysis

To assess cell numbers more intuitively, Calcein-AM (Beyotime, Shanghai, China) was first diluted to the correct concentration, after which it was incubated with the cells at 37 °C and 5% CO_2_ for 30 min. Fluorescence images of each well were then captured using the ImageXpress Pico automated cell imaging analysis system (Molecular Devices, San Jose, CA, USA) at 10× magnification. Image segmentation and counting were then performed to generate the final statistical output of cell counts.

### PDNVs uptake essay

After 30 min of incubation at 37 °C, the PDNVs were labeled with DiI dye (Beyotime, Shanghai, China). Subsequently, the stained PDNVs were co-incubated with H9c2 cells for 6 h. Following this incubation, the H9c2 cells were stained with FITC-labeled phalloidin (Solarbio, Beijing, China) according to the manufacturer’s protocol and were finally visualized under a confocal laser scanning microscope (Leica, Wetzlar, Germany).

### H_2_O_2_ scavenging assay

To assess whether PDNVs directly scavenge H_2_O_2_, the residual H_2_O_2_ level was measured using the Amplex Red assay. Three experimental groups were set up: blank control (DMEM only), model group (DMEM containing 600 μmol·L^-1^ H_2_O_2_), and treatment group (DMEM containing 600 μmol·L^-1^ H_2_O_2_ and 40 μg·mL^-1^ PDNVs). All samples were incubated at 37 °C for 0, 0.5, 1, 2, and 4 h. At each time point, aliquots were collected and processed as recommended. Absorbance at 570 nm (OD_570_) was measured using a microplate reader to indicate the relative H_2_O_2_ content.

### Determination of DPPH· inhibition rate for glu-PDNVs

50 µL of the Glu-PDNVs test solution was mixed with 50 µL of the 0.200 mmol·L^-1^ DPPH· ethanol solution (Macklin, Shanghai, China) in a 200 µL tube. The mixture was thoroughly agitated on a vortex mixer, then left to stand in darkness at ambient temperature for 30 min, after which its absorbance at 517 nm was determined and recorded as A_i_. Similarly, the absorbance (A_0_) was determined by mixing 50 µL of PBS with 50 µL of the DPPH· solution, and the absorbance (A_j_) was determined by mixing 50 µL of the Glu-PDNVs test solution with 50 µL of anhydrous ethanol. The Scavenging rate (S) of the Glu-PDNVs against the DPPH· was determined by the following formula^[42]^:

**Figure:**
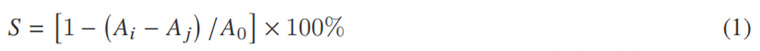


### Evaluation of oxidative stress

To evaluate the efficacy of PDNVs in suppressing H_2_O_2_-induced ROS accumulation in cardiomyoblasts, cells were stained using the ROS Assay Kit (Beyotime, Shanghai, China) according to the protocol, and then analysed by flow cytometry (Beckman, Brea, CA, USA).

To evaluate the level of superoxide ions within the mitochondria, cells were stained with MitoSOX and MitoTracker (Beyotime, Shanghai, China) according to the instructions provided. Finally, multi-channel images were acquired using confocal laser scanning microscopy^[43]^.

The activities of SOD and GPx were evaluated using commercial assay kits (Beyotime, Shanghai, China) according to the manufacturer’s instructions. Total protein concentration was determined by BCA assay to normalize the activities. SOD and GPx activities were expressed as U/mg protein.

### Statistical analysis

We performed one-way ANOVA with GraphPad Prism 9 (San Diego, CA, USA) to analyze the data among multiple groups. Data are presented as mean ± SD. Differences with *P* < 0.05 were interpreted as statistically significant. Figure preparation was supported by the following software: ImageJ, FlowJo, and Adobe Illustrator.

## RESULTS

### Characterization and cellular uptake of PDNVs

**Figure 1:**
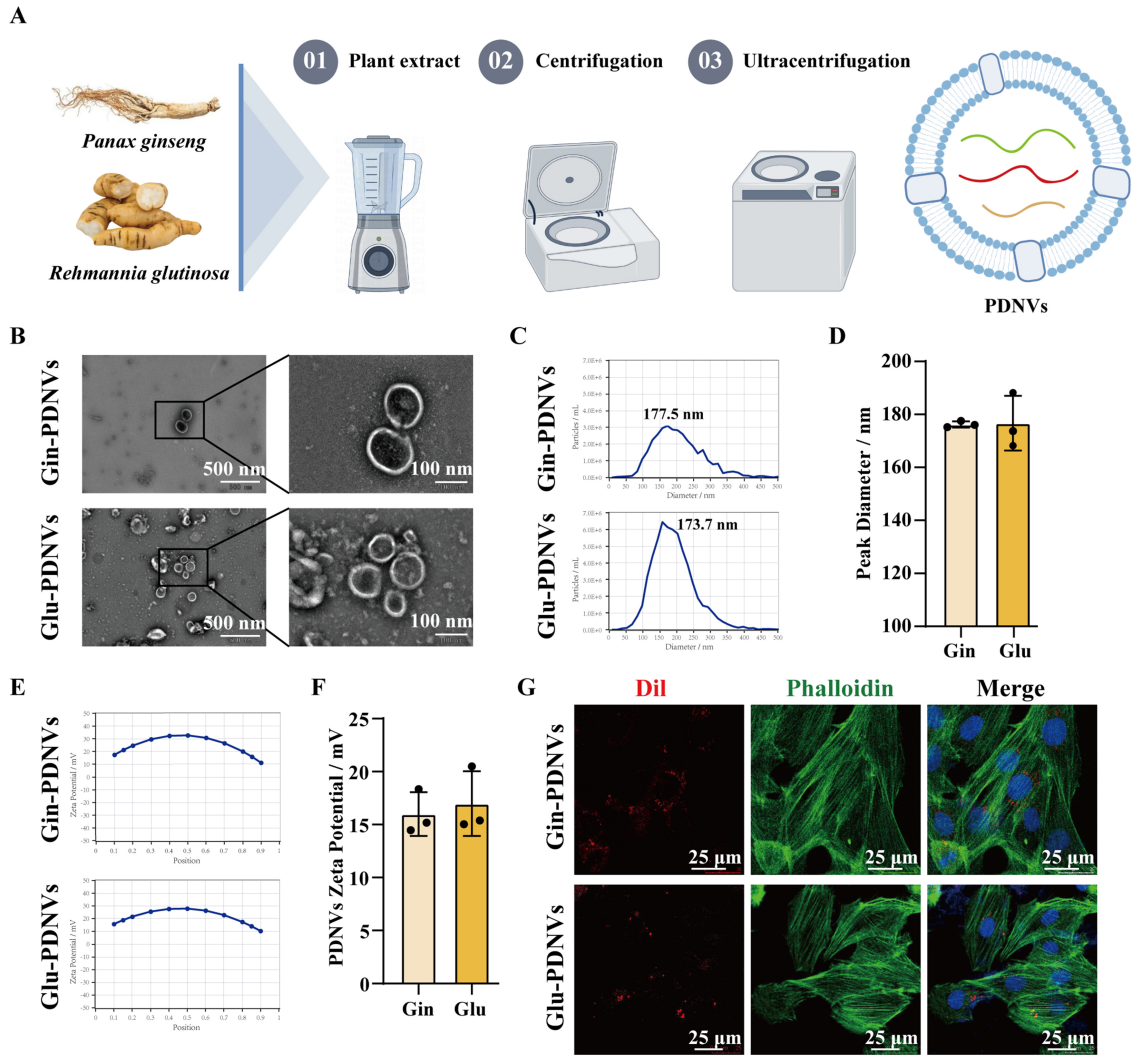
Characterization and cellular uptake of PDNVs. (A) Isolation of PDNVs from Gin and Glu by differential ultracentrifugation; (B) TEM images of Gin-PDNVs and Glu-PDNVs (Scale bars = 500 and 100 nm); NTA analysis of (C and D) diameter distribution and (E and F) zeta potential of Gin-PDNVs and Glu-PDNVs. *n* = 3; (G) DiI (red)-labelled PDNVs in phalloidin (green)-labelled H9c2 cells (Scale bar = 25 µm). Data are presented as mean ± SD. PDNVs: Plant-derived nanovesicles; Gin: *Panax ginseng*; Glu: *Rehmannia glutinosa*; TEM: transmission electron microscopy; NTA: nanoparticle tracking analysis; DiI: 1,1’-dioctadecyl-3,3,3’,3’-tetramethylindocarbocyanine perchlorate; SD: standard deviation; EVs: extracellular vesicles; nm: nanometer; µm: micrometer; mV: millivolt.

Ultracentrifugation was employed in this study to extract PDNVs from Gin and Glu, and these PDNVs were characterised using TEM, NTA and zeta potential analysis [Figure 1A]. TEM images revealed that Gin-PDNVs and Glu-PDNVs exhibited a typical cup-shaped morphology with intact bilayer membrane structures, resembling animal-derived EVs [Figure 1B, scale bars: 500 and 100 nm]. NTA was used to determine the particle concentration and size distribution, showing that the diameters of the two types of PDNVs were mainly distributed between 30-200 nm [Figure 1C and D]. Meanwhile, zeta potential measurements further indicated that these PDNVs possessed good stability [Figure 1E and F]. Table 1 summarizes the relevant parameters of Gin-PDNVs and Glu-PDNVs.

**Table 1:** Corresponding parameters of PDNVs

**Parameter**	**Gin**		**Glu**	
	**Original**	**Normalized**	**Original**	**Normalized**
Plant material (g)	50	50	250	50
Resuspension vol. (mL)	7.5	5	10.0	5
Protein conc. (µg·mL^-1^)	4,341	6,511.5	4,971.7	1,988.7
Particle conc. ( 10^11^·mL^-1^)	3.3	5.0	15	6.0
Protein yield (µg·g^-1^)	651.2		198.9	
Particles/protein (× 10^7^·µg^-1^)	7.6		30	
Particle yield (× 10^10^·g^-1^)	4.9		6.0	
Size (nm)	176.2		176.7	
Zeta (mV)	16		17	

PDNVs: Plant-derived nanovesicles; Gin: *Panax ginseng*; Glu: *Rehmannia glutinosa*; vol.: volume; conc.: concentration; µg: microgram; mL: milliliter; g: gram; nm: nanometer; mV: millivolt.

To investigate the cellular uptake of PDNVs by H9c2 cells, DiI-labeled PDNVs were co-incubated with H9c2 cells for 6 h, after which the cytoskeleton was stained with phalloidin. Confocal microscopy revealed that DiI-labeled Gin-PDNVsand Glu-PDNVs (red spots) were all localised within the cytoskeleton [Figure 1G]. This indicates that PDNVs from these two different sources can be efficiently internalised by H9c2 cells.

### Protective effects of Gin-PDNVs on H_2_O_2_-injured H9c2 cells

**Figure 2:**
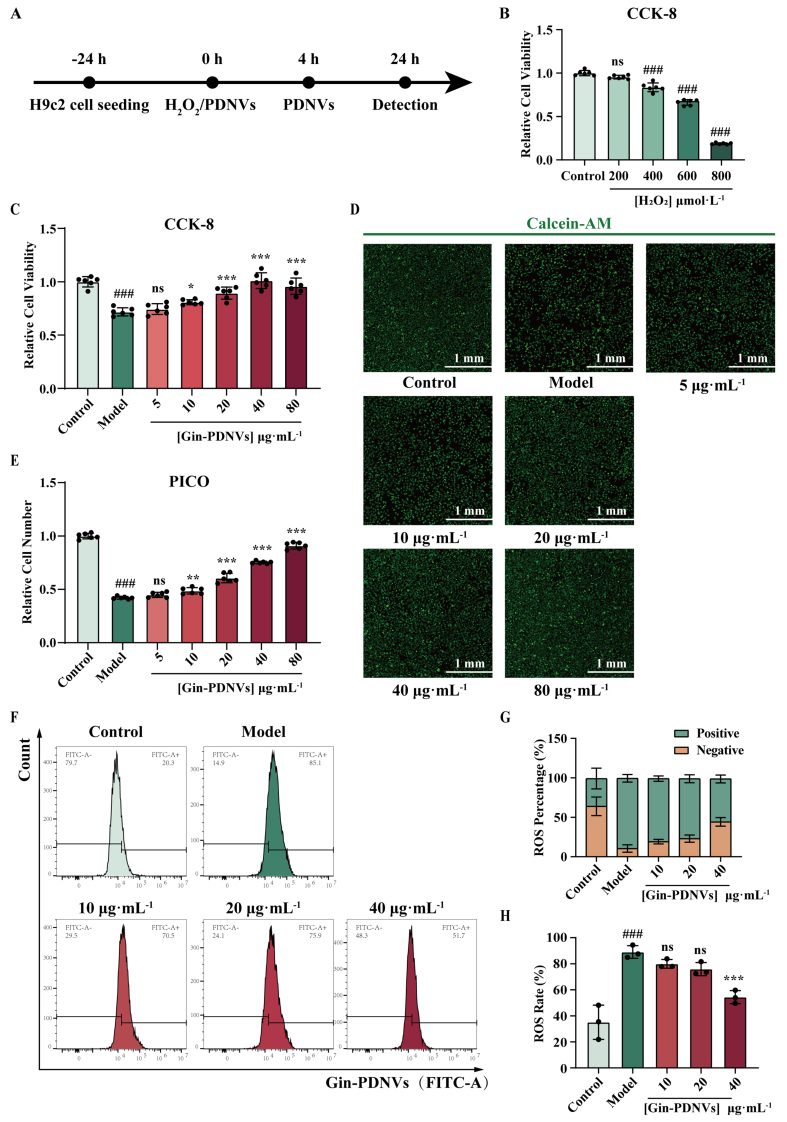
The potent cytoprotective properties of Gin-PDNVs on H9c2 cells against H_2_O_2_-induced injury. (A) Schematic illustration outlining the experimental workflow; (B) Relative viability of H9c2 cells following 4 h exposure to 200, 400, 600 and 800 µmol·L^-1^ H_2_O_2_. *n* = 6; (C) Relative viability of H9c2 cells after Gin-PDNVs treatment. *n* = 6; (D) Calcein-AM (green)-labeled H9c2 cells after Gin-PDNVs treatment (Scale bar = 1 mm); (E) Relative number of H9c2 cells from (D). *n* = 6; (F) ROS levels in H9c2 cells were measured by flow cytometry; (G) Quantitative analysis of ROS-positive and -negative cell populations. *n* = 3; (H) Representative flow cytometry histograms of intracellular ROS levels. *n* = 3. Data are presented as mean ± SD and analyzed by one-way ANOVA (^###^*P* < 0.001 *vs.* control; ^*^P < 0.05, ^**^P < 0.01, ^***^P < 0.001 *vs.* model). PDNVs: Plant-derived nanovesicles; Gin-PDNVs: *Panax ginseng*-derived nanovesicles; H_2_O_2_: hydrogen peroxide; CCK-8: cell counting kit-8; Calcein-AM: calcein acetoxymethyl ester; PICO: ImageXpress Pico; ROS: reactive oxygen species; FITC: fluorescein isothiocyanate; ANOVA: analysis of variance; SD: standard deviation; mm: millimeter; µmol: micromole; µg: microgram; mL: milliliter; L: liter.

To assess the cytoprotective function of PDNVs, we evaluated the viability of H9c2 cells subjected to H_2_O_2_-induced injury [Figure 2A]. The viability of H9c2 cells exposed to H_2_O_2_ at gradient concentrations (200, 400, 600, and 800 μmol·L^-1^) was measured using the CCK-8 assay [Figure 2B]. Results showed that cell viability gradually decreased with increasing H_2_O_2_ concentration. At 400 μmol·L^-1^ H_2_O_2_, cell viability was significantly reduced compared to the control group and further declined to approximately 70% at 600 μmol·L^-1^, a concentration that was selected for subsequent experiments.

In the H9c2 injury model induced by 600 μmol·L^-1^ H_2_O_2_, cells were treated with Gin-PDNVs at various concentrations (5, 10, 20, 40, and 80 μg·mL^-1^). CCK-8 assay revealed that cell viability was significantly inhibited in the model group compared to the control group [Figure 2C]. In contrast, Gin-PDNVs at 10 μg·mL^-1^ demonstrated a modest protective effect compared to the model group (*P* = 0.0354), while concentrations of 20, 40, and 80 μg·mL^-1^ exhibited strong protective effects (*P* < 0.001).

To further visually assess changes in cell number, live cells were stained with Calcein-AM and analyzed using the ImageXpress Pico automated imaging and analysis system. Representative images showed that, relative to the control, the model group had markedly fewer cells, and this reduction was gradually reversed by increasing concentrations of Gin-PDNVs [Figure 2D]. Quantitative analysis was consistent with the trends observed in the CCK-8 assay [Figure 2E].

To further validate the cytoprotective effects of Gin-PDNVs against H_2_O_2_-induced oxidative stress in H9c2 cells, we employed flow cytometry to assess ROS accumulation following treatment with Gin-PDNVs at varying concentrations (10, 20, and 40 µg·mL^-1^). The ROS assay showed that the model group exhibited a significant increase in ROS levels [Figure 2F-H; *P* < 0.001 *vs.* control]. In contrast, following treatment with 40 µg·mL^-1^ Gin-PDNVs, intracellular ROS levels were significantly reduced (*P* = 0.0005). These results demonstrate that Gin-PDNVs exert potent cytoprotective effects against H_2_O_2_-induced injury in H9c2 cells.

### Protective effects of Glu-PDNVs on H_2_O_2_-injured H9c2 cells

**Figure 3:**
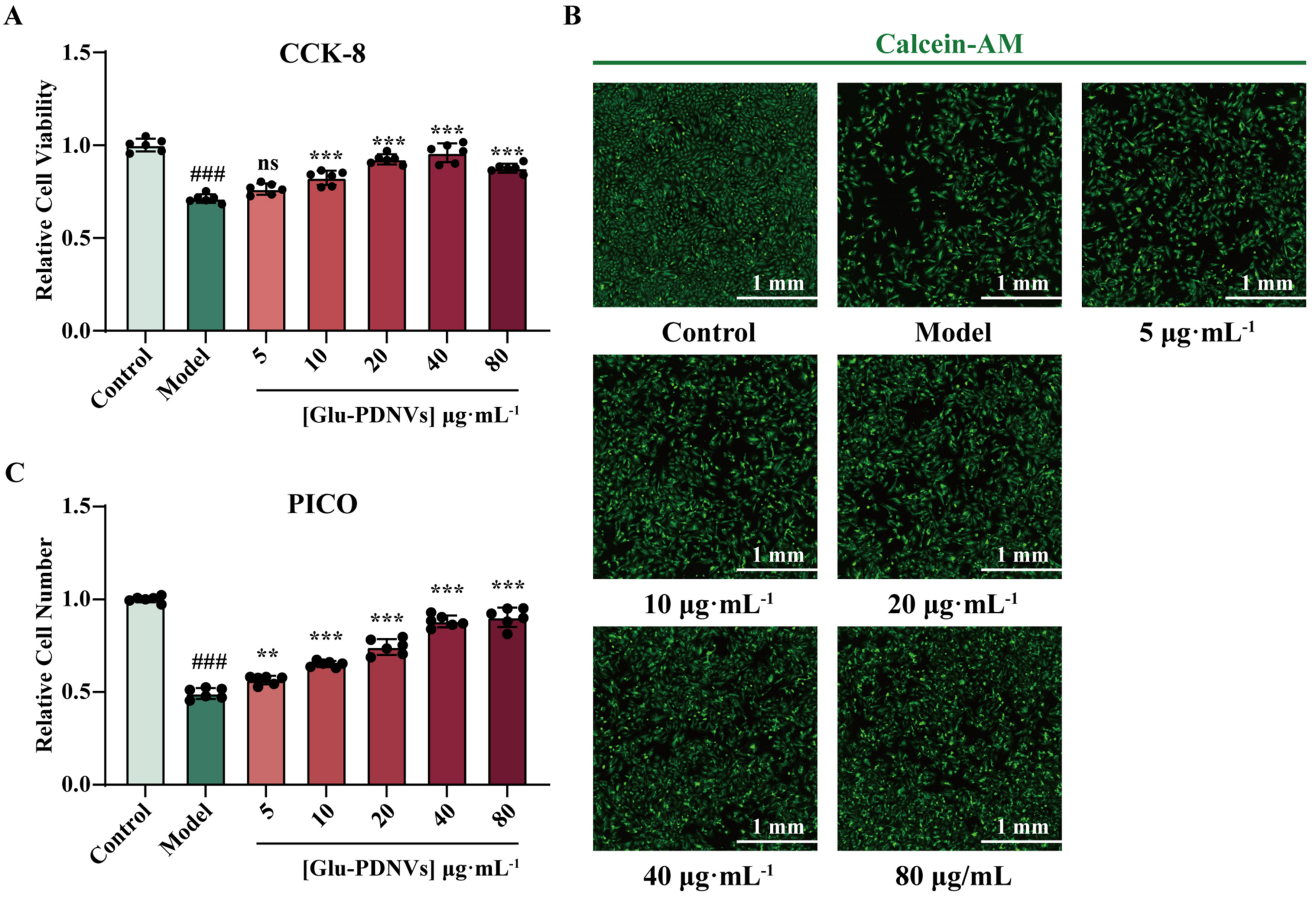
The potent cytoprotective properties of Glu-PDNVs on H9c2 cells against H_2_O_2_-induced injury. (A) Relative viability of H9c2 cells after Glu-PDNVs treatment. *n* = 6; (B) Calcein-AM (green)-labeled H9c2 cells after Glu-PDNVs treatment (Scale bar = 1 mm); (C) Relative number of H9c2 cells from (B). *n* = 6. Data are presented as mean ± SD and analyzed by one-way ANOVA (^###^*P* < 0.001 *vs.* control; ^**^P < 0.01, ^***^P < 0.001 *vs.* model). PDNVs: Plant-derived nanovesicles; Glu-PDNVs: *Rehmannia glutinosa*-derived nanovesicles; H_2_O_2_: hydrogen peroxide; CCK-8: cell counting kit-8; Calcein-AM: calcein acetoxymethyl ester; PICO: ImageXpress Pico; ANOVA: analysis of variance; SD: standard deviation; mm: millimeter; µmol: micromole; µg: microgram; mL: milliliter; L: liter.

The efficacy of Glu-PDNVs was similarly evaluated. CCK-8 assay revealed that cell viability was significantly inhibited in the model group compared to the control group [Figure 3A]. In contrast, Glu-PDNVs treatment at concentrations of 10, 20, 40, and 80 µg·mL^-1^ resulted in significant protective effects (*P* < 0.001).

Automated cell imaging further confirmed these findings [Figure 3B]. The model group exhibited a clear decrease in cell number relative to the control, while Glu-PDNVs treatment led to a concentration-dependent increase in cell density. Quantitative analysis of these images yielded results consistent with the CCK-8 assay [Figure 3C].

### Compositional analysis of Glu-PDNVs

**Figure 4:**
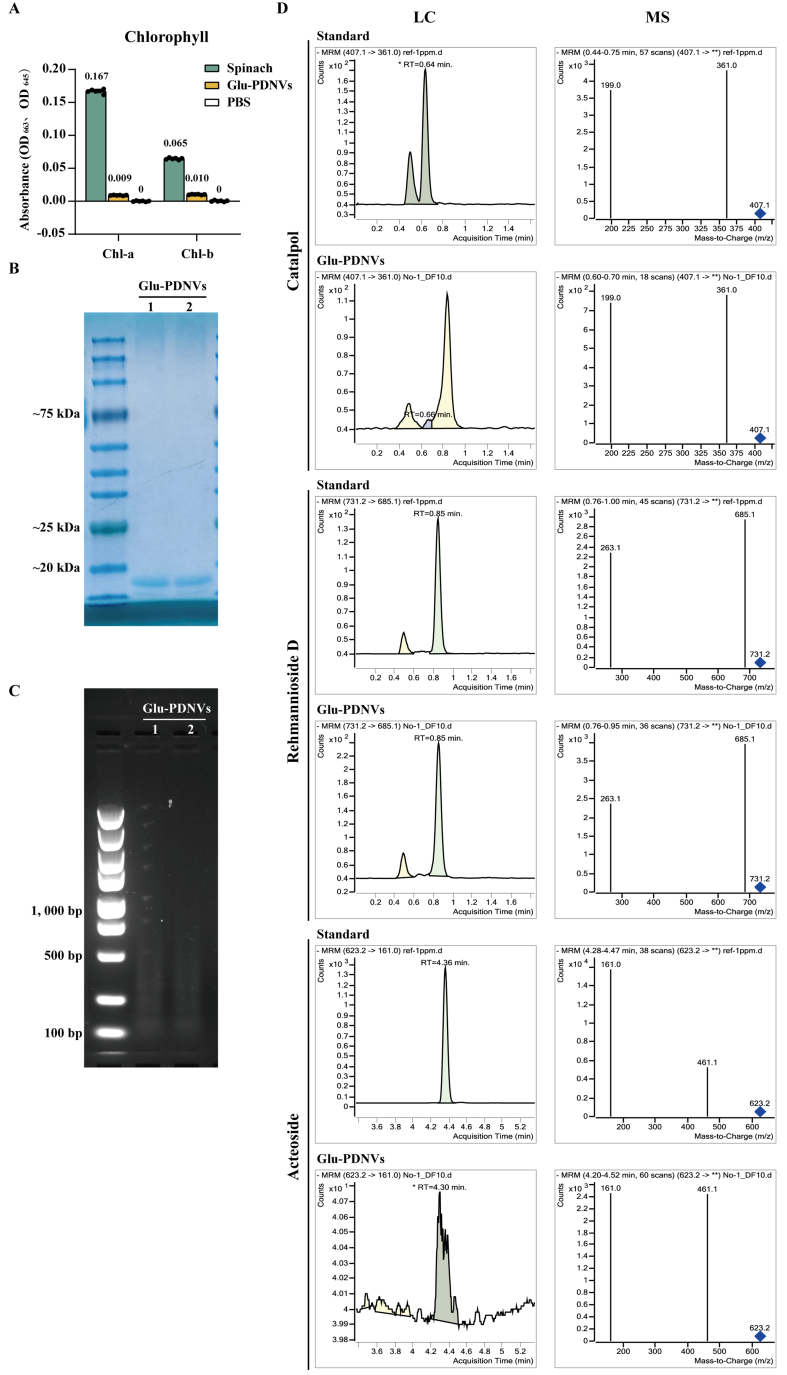
Compositional Analysis of Glu-PDNVs. (A) Chlorophyll content analysis of Glu-PDNV preparations. Absorbance was measured at 663 and 645 nm, the characteristic wavelengths for chlorophyll a and b, respectively. *n* = 3. Data are presented as mean ± SD; (B) Protein composition of Glu-PDNVs was evaluated by Coomassie Blue staining; (C) Nucleic acid composition was analyzed by agarose gel electrophoresis; (D) Representative LC-MS chromatograms for catalpol, rehmannioside D, and acteoside in Glu-PDNVs. PDNVs: Plant-derived nanovesicles; Glu-PDNVs: Rehmannia glutinosa-derived nanovesicles; LC-MS: liquid chromatography-mass spectrometry; MRM: multiple reaction monitoring; RT: retention time; m/z: mass-to-charge ratio; OD: optical density; Chl-a: chlorophyll a; Chl-b: chlorophyll b; PBS: phosphate-buffered saline; SD: standard deviation; kDa: kilodalton; bp: base pair.

To assess chloroplast contamination, chlorophyll content was measured. PDNVs showed minimal absorbance at 663 and 645 nm (OD ≈ 0.01), over 18-fold lower than the spinach homogenate control (OD_663_ = 0.167), indicating effective removal of chloroplast-derived material during purification [Figure 4A]. The presence of both protein and nucleic acid cargo within Glu-PDNVs was confirmed through Coomassie Brilliant Blue staining and agarose gel electrophoresis, respectively [Figure 4B and C]. Glu-PDNVs exhibited a predominant protein band at approximately 10 kDa on SDS-PAGE and were found to contain nucleic acids at a concentration of 3,789.3 ng·mL^-1^ by NanoDrop spectrophotometry. To determine whether Glu-PDNVs selectively enrich the bioactive constituents of Glu, we performed LC-MS analysis targeting three representative compounds-catalpol, rehmannioside D and acteoside-all of which possess well-documented antioxidant activity[Figure 4D]^[34-36,44]^. The absolute concentrations of these compounds and nucleic acid, as well as their values normalized to particle count and protein concentration, are presented in Table 2.

**Table 2:** Normalized nucleic acid and bioactive compound contents of Glu-PDNVs

	**Nucleic acid**	**Catalpol**	**Rehmannioside D**	**Acteoside**
**Original concentration (g·mL^-1^)**	3.79	0.29	21.91	0.01
**Per 10^9^ particles (ng)**	0.25	0.02	1.46	8.67 10^-4^
**Per μg protein (ng)**	0.76	0.06	4.41	2.61 10^-3^
**Concentration at 40 ug/mL protein (ug·mL^-1^)**	0.03	2.3310^-3^	0.18	1.05 10^-4^

PDNVs: Plant-derived nanovesicles; Glu-PDNVs: *Rehmannia glutinosa*-derived nanovesicles; µg: microgram; ng: nanogram; mL: milliliter; g: gram.

### Glu-PDNVs protect H9c2 cells against H_2_O_2_-induced oxidative injury

**Figure 5:**
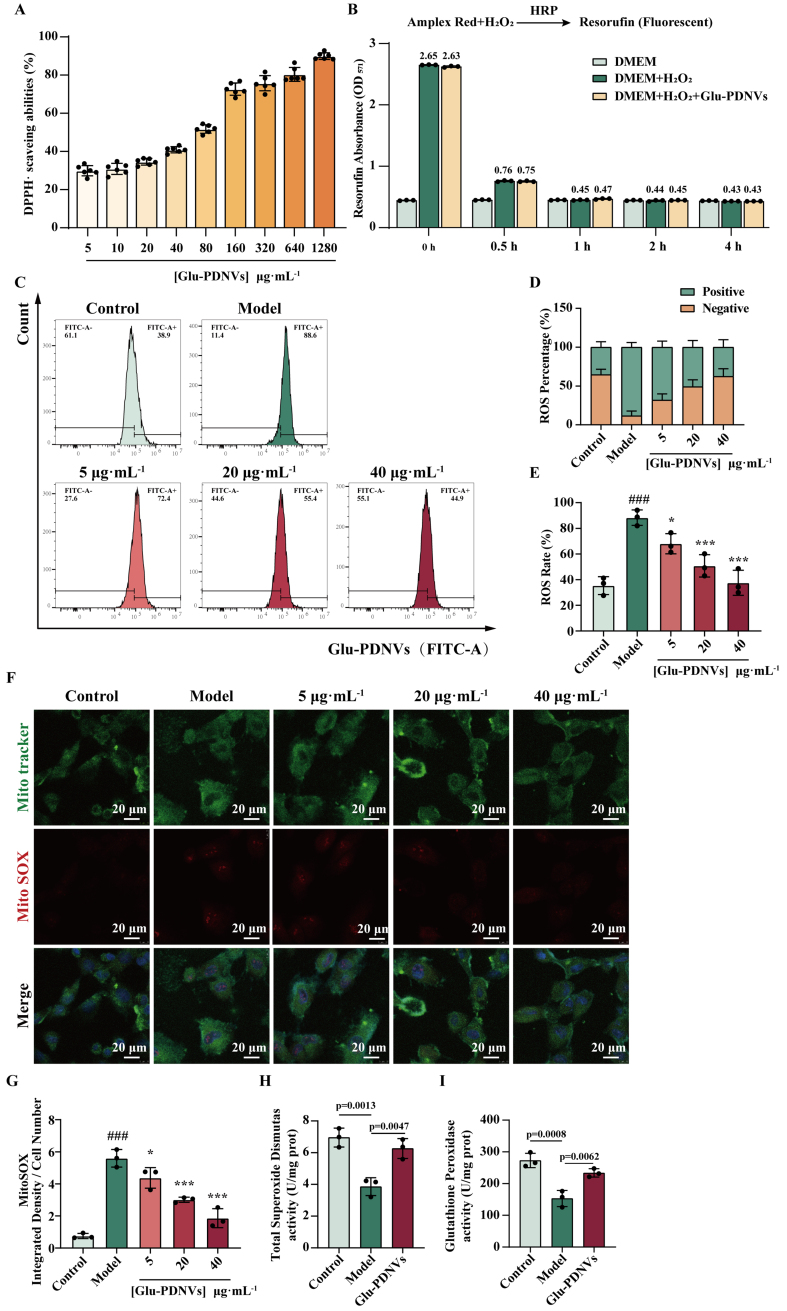
Glu-PDNVs effectively protect H9c2 cells from oxidative damage caused by H_2_O_2_. (A) The inhibition rate of Glu-PDNVs against DPPH· radicals at gradient concentrations. *n* = 6; (B) Direct H_2_O_2_ scavenging capacity of Glu-PDNVs. *n* = 3; (C) ROS levels in H9c2 cells were quantified by flow cytometry; (D) Quantitative analysis of ROS-positive and -negative cell populations; (E) Representative flow cytometry histograms of intracellular ROS levels. *n* = 3; (F) Representative fluorescence images and (G) statistical analysis of MitoTracker and MitoSOX staining in H9c2 cells. *n* = 3; Effects of Glu-PDNVs on endogenous antioxidant enzyme activities in H_2_O_2_-injured H9c2 cells, showing (H) SOD activity and (I) GPx activity. *n* = 3. Data are presented as mean ± SD and analyzed by one-way ANOVA (^###^*P* < 0.001 *vs.* control; ^*^P < 0.05, ^***^P < 0.001 *vs.* model). PDNVs: Plant-derived nanovesicles; Glu-PDNVs: *Rehmannia glutinosa*-derived nanovesicles; H_2_O_2_: hydrogen peroxide; HRP: horseradish peroxidase; Amplex Red: 10-acetyl-3,7-dihydroxyphenoxazine; DMEM: Dulbecco’s Modified Eagle Medium; DPPH·: 2,2-diphenyl-1-picrylhydrazyl radical; ROS: reactive oxygen species; FITC: fluorescein isothiocyanate; MitoTracker: mitochondrial tracker dye; MitoSOX: mitochondrial superoxide indicator; SOD: superoxide dismutase; GPx: glutathione peroxidase; OD: optical density; ANOVA: analysis of variance; SD: standard deviation; µg: microgram; mL: milliliter; µm: micrometer.

Measurement of the DPPH· inhibition rate indicates that PDNVs exhibit significant antioxidant activity, which increases with concentration [Figure 5A]. To exclude the possibility that PDNVs directly quench H_2_O_2_, we measured H_2_O_2_ consumption using the Amplex Red fluorescence assay. Results showed that the difference in OD values between the PDNV-treated group and the H_2_O_2_-only group remained minimal (≤ 0.03 at all time points), indicating no substantial direct scavenging effect [Figure 5B]. To further assess the cytoprotective role of Glu-PDNVs against oxidative stress induced by H_2_O_2_ in H9c2 cells, we measured intracellular ROS and superoxide levels following treatment with gradient concentrations of PDNVs (5, 20, and 40 µg·mL^-1^).

Flow cytometry analysis of intracellular ROS levels showed a significant increase in the model group [Figure 5C-E; *P* < 0.001 *vs.* control]. In contrast, Glu-PDNVs reduced ROS levels in a concentration-dependent manner, with significant inhibition observed at 5 (*P* = 0.0339), 20 (*P* = 0.0006), and 40 µg·mL^-1^ (*P* < 0.0001). Furthermore, compared to Gin-PDNVs, Glu-PDNVs exhibited a more pronounced inhibitory effect on intracellular ROS.

Mitochondrial superoxide (MitoSOX), the primary superoxide anion serves as a key molecular marker of oxidative stress. Confocal analysis demonstrated that MitoSOX fluorescence intensity was markedly higher in the model group than in controls under identical cell number conditions. This elevation was dose-dependently suppressed by Glu-PDNVs, reaching significance at concentrations of 20 and 40 µg·mL^-1^ [Figure 5F and G].

To further investigate the mechanisms underlying the cytoprotective effects of Glu-PDNVs, we examined the activities of key antioxidant enzymes SOD and GPx. Results showed that H_2_O_2_ treatment significantly reduced SOD activity in H9c2 cells (*P* < 0.01 *vs.* control). whereas 40 μg·mL^-1^ Glu-PDNVs significantly restored SOD activity [Figure 5H]. Similarly, GPx activity was markedly suppressed by H_2_O_2_ exposure and was significantly elevated upon Glu-PDNVs treatment [Figure 5I]. These results demonstrate that Glu-PDNVs enhance the cellular antioxidant defense capacity not only through direct radical scavenging but also by upregulating the activity of endogenous antioxidant enzymes.

## DISCUSSION

The present study demonstrates, for the first time, that Glu-PDNVs possess significant antioxidant and cardioprotective effects against H_2_O_2_-induced oxidative injury in H9c2 cardiomyoblasts. Comparative analysis revealed that Glu-PDNVs exhibited protective effects slightly superior to those of Gin-PDNVs under equivalent experimental conditions. Notably, NTA showed that at the same protein concentration (40 μg·mL^-1^), Glu-PDNVs had a higher particle count than Gin-PDNVs [Table 1]. This higher vesicle yield per unit protein may contribute to their enhanced efficacy. Mechanistically, Glu-PDNVs effectively attenuated oxidative stress-induced cellular damage, as evidenced by significantly reduced intracellular ROS accumulation and MitoSOX levels. These findings suggest that Glu-PDNVs enhance cellular antioxidant defense capacity, consistent with the observed restoration of SOD and GPx activities.

Beyond their bioactivity, Glu-PDNVs offer practical advantages for translational development. Glu has a short growth cycle with well-established cultivation techniques and high biomass yield, enabling cost-effective and scalable production. In contrast, Gin requires 4-6 years of growth before harvest, which limits its economic feasibility for large-scale vesicle production. The combination of potent antioxidant activity and superior production efficiency positions Glu-PDNVs as a promising and sustainable nanotherapeutic platform for myocardial protection.

Unlike animal CDVs, PDNVs not only contain fundamental components such as lipids, proteins, and nucleic acids, but are also rich in various pharmacologically active natural phytochemicals. Gin-PDNVs exhibit antioxidant activity with their known content of multiple ginsenosides, e.g. Rg1, Re, Rg3, and Rb1, all of which were reported to possess antioxidant activity^[45,46]^. In this study, LC-MS analysis identified catalpol, rehmannioside D and acteoside in Glu-PDNVs. Rehmannioside D and catalpol both belong to the iridoid glycosides class. Previous studies have demonstrated that both compounds exhibit potent antioxidant activity in the PC-12 cell model, and catalpol has been shown to mitigate oxidative stress in myocardial ischemia-reperfusion injury^[35,47]^. Rehmannioside D shares structural and functional similarities with catalpol, suggesting it may also exert antioxidative stress effects in myocardial tissue. Furthermore, its high enrichment within Glu-PDNVs indicates that rehmannioside D could be a key component mediating the effects of Glu-PDNVs, though its specific pharmacological efficacy requires further validation. While these findings highlight rehmannioside D as a potential key contributor, it is important to emphasize that the cardioprotective effect of Glu-PDNVs is unlikely to result from a single compound alone. We identify catalpol, rehmannioside D, and acteoside as quantifiable, characteristic chemical tracers, demonstrating that PDNVs naturally encapsulate bioactive constituents from their parent plant. However, PDNVs also contain other bioactive molecules, including peptides, lipids, and additional small-molecule compounds, which could synergistically contribute to the overall therapeutic effect.

Despite these promising findings, several limitations of the present study should be acknowledged. First, while PDNVs function as integrated nanotherapeutic systems with synergistic multi-component interactions, the relative contribution of individual compounds to the observed effects remains unclear. Future studies testing the biological activity of purified compounds at physiologically relevant levels, both *in vitro* and *in vivo*, will help clarify this question. Second, the use of H9c2 cells, a rat cardiac myoblast cell line, limits the translational relevance of the results. While H9c2 cells are well-suited for preliminary mechanistic studies due to their ease of culture and rapid proliferation, they differ from human cardiomyocytes in cellular phenotype, physiological function, and signaling pathways. Future studies should use models that better mimic human cardiac physiology, for example, human induced pluripotent stem cell-derived cardiomyocytes. Third, as this study was performed entirely *in vitro*, the effects of Glu-PDNVs were not evaluated in *in vivo* cardiovascular models. Animal models of myocardial ischemia-reperfusion injury may be used to further assess the translational potential of Glu-PDNVs. Finally, while we have identified and quantified several characteristic compounds in Glu-PDNVs, it is not yet determined which of these components are primarily responsible for the observed cardioprotective effects. Understanding the material basis of their bioactivity will be essential for future optimization and development of Glu-PDNVs for cardiovascular applications.

In conclusion, this study provides the first evidence that Glu-PDNVs possess significant antioxidant and cardioprotective effects against oxidative stress in H9c2 cardiomyoblasts. Mechanistically, Glu-PDNVs attenuated cellular damage by reducing intracellular ROS and MitoSOX levels, while restoring the activities of key antioxidant enzymes, SOD and GPx. Their superior efficacy compared to Gin-PDNVs under equivalent conditions may be attributed to a higher vesicle yield per unit protein and the enrichment of bioactive compounds, particularly rehmannioside D. While these findings establish Glu-PDNVs as a promising and sustainable nanotherapeutic platform derived from Glu, further investigations using *in vivo* models and human-derived cardiomyocytes are necessary to validate their translational potential for myocardial protection.

## DECLARATIONS

### Availability of data and materials

All relevant data are included within the paper. The data that support the findings of this study are available from the corresponding author upon reasonable request.

### AI and AI-assisted tools statement

During the preparation of this manuscript, the AI tool DeepL Write and DeepSeek (version V3.2, released 2025-12-01) were used solely for language editing. The tool did not influence the study design, data collection, analysis, interpretation, or the scientific content of the work. All authors take full responsibility for the accuracy, integrity, and final content of the manuscript.

### Copyright

© The Author(s) 2026.
